# Correlation of renal biomarkers, electrolyte imbalances and vitamin D levels in hypertensive subjects

**DOI:** 10.3892/mi.2025.219

**Published:** 2025-02-05

**Authors:** Adeola O. Oluboyo, Emmanuel A. Omon, Bernard O. Oluboyo, Odeyinka O. Odewusi, Obongama O. Edet

**Affiliations:** Department of Medical Laboratory Science, College of Medicine and Health Sciences, Afe Babalola University, P.M.B. 5454, Ado-Ekiti, Ekiti State, Nigeria

**Keywords:** high blood pressure, hypertension, electrolyte imbalance, renal function, vitamin D

## Abstract

The mechanisms behind persistent high blood pressure and its effects on renal function and electrolyte balance are not yet fully understood. The present study aimed to identify electrolyte imbalances in hypertensive patients and to determine their association with renal function and vitamin D levels. For this purpose, 155 subjects were recruited for the study, including 83 hypertensive subjects and 72 healthy normotensive subjects matched for sex and age as controls. Demographic data were collected using a questionnaire, and anthropometric parameters were measured using standard procedures. Sodium and potassium levels were determined using an ion selective electrolyte analyzer, while calcium, magnesium and phosphorus levels were measured using spectrophotometry. Uric acid, creatinine and urea levels were analyzed using a semi-auto chemistry analyzer, and vitamin D levels were assessed using ELISA. The results obtained revealed that body mass index, systolic and diastolic blood pressure, and creatinine, urea, uric acid, magnesium and sodium levels were significantly higher in the hypertensive subjects compared with the healthy controls (P<0.05); however, the vitamin D, calcium, potassium and phosphate levels were significantly lower in the hypertensive subjects compared to the healthy controls (P<0.05). There was no statistically significant difference in all parameters studied as regards age and sex (P>0.05). On the whole, the present study demonstrates that an electrolyte imbalance, renal dysfunction and vitamin D deficiency are observed in hypertensive subjects. These findings emphasize the importance of assessing and monitoring these biochemical markers as they could improve prognosis, aid in early diagnosis, and assist in determining the optimal level of therapeutic interventions.

## Introduction

Hypertension is a medical condition in which the pressure of blood in the arteries is consistently elevated. Of note, 15-20% of adults worldwide are affected by hypertension, which is defined as having a systolic blood pressure of at least 140 mmHg and/or a diastolic blood pressure of at least 90 mmHg (1). Although high blood pressure typically does not exhibit any symptoms, it can lead to severe health issues, such as peripheral arterial disease, atrial fibrillation, stroke, heart failure, chronic kidney disease, vision loss and dementia (2). Hypertension is a leading cause of premature mortality globally. High blood pressure is divided into primary or essential hypertension and secondary hypertension. Of note, 90-95% of cases are primary, characterized by high blood pressure caused by non-specific lifestyle and genetic factors (3). Risk factors for primary hypertension include excessive salt consumption, obesity, smoking, a lack of physical activity and alcohol consumption. The remainder 5-10% of cases fall under secondary hypertension, which is high blood pressure caused by a specific identifiable factor, such as chronic kidney disease, kidney artery narrowing, an endocrine disorder, or the use of birth control pills (4).

Electrolytes are compounds that play a crucial role in various bodily functions. They help maintain the balance of fluids inside and outside cells, as well as contribute to muscle contraction and heart function. The evaluation of electrolyte levels has been a focus of research worldwide for a number of years. Any imbalance in electrolytes can disrupt normal bodily functions, leading to abnormal increases or decreases in levels (5). Electrolyte imbalances are often the primary indicator of numerous diseases and are prevalent in both the general population and hospitalized patients. Previous research has indicated that electrolyte disorders are linked to higher rates of morbidity and mortality (6). Imbalances in electrolyte levels can also contribute to the development of severe cardiovascular conditions, such as hypertension. It is crucial to investigate whether electrolyte imbalances are connected to specific risk factors for chronic diseases that can be life-threatening (7). Understanding this association can help in the management of these risk factors by regulating serum electrolyte levels. This proactive approach may aid in the prevention or delay in the onset of these diseases and their associated complications, particularly in cases of hypertension.

Clinical investigations of renal function, including uric acid, urea and creatinine levels, are essential for identifying renal dysfunction, diagnosing renal disease, monitoring disease progression, and assessing response to treatment. The relative risk of severe renal damage in patients with uncomplicated essential hypertension is low compared to other cardiovascular complications (8). However, due to the high prevalence of hypertension in the general population, it remains the second leading cause of end-stage renal disease, with the risk being significantly higher in individuals of African origin. Renal dysfunction is recognized as an independent risk factor for morbidity and mortality in congestive heart failure. The well-established association between heart failure and chronic kidney disease is known as the cardiorenal syndrome (9). It has been shown that renal dysfunction is independently associated with the development of heart failure in hypertensive patients. Hypertension and renal impairment have a bidirectional cause-and-effect association, with each being a common cause of the other. Therefore, the early diagnosis of kidney disease is crucial for effectively managing patients with hypertension (10).

Vitamin D is a prohormone that is converted metabolically to its active form, 1,25-dihydroxyvitamin D [1,25(OH)2D]. This hormone activates the vitamin D receptor, which then regulates the transcription of genes responsible for various biological responses (11). Vitamin D is obtained through the diet and exposure to sunlight, and plays a crucial role in regulating calcium levels in the body. It influences the absorption and metabolism of calcium by influencing voltage-dependent calcium channels and the renin-angiotensin-aldosterone system (RAAS). This system controls renin production, which in turn affects the secretion of parathyroid hormone (12). Research conducted in recent years has revealed a number of other biological functions of vitamin D, including cell differentiation, cell growth inhibition, immunomodulation and the regulation of hormonal systems (7). Additionally, vitamin D has been shown to improve endothelial function by increasing nitric oxide production, preventing calcification, maintaining vascular tone and reducing inflammation. Furthermore, the lack of vitamin D has been linked to the development of non-skeletal conditions, such as hypertension, kidney disease, and insulin resistance (13). Therefore, the present study was performed to determine the mechanisms through which electrolyte disturbances in hypertensive patients are associated with renal function and vitamin D levels in a Nigerian hypertensive population.

## Subjects and methods

### Study area

The present study was conducted at the Federal Teaching Hospital Ido (FETHI), Ido-Ekiti, Ekiti State. Ekiti State is primarily a highland area located ~50 m above sea level, with coordinates of 7˚40'N 5˚15'E. It is bordered to the north by Kwara State for 61 km, to the northeast by Kogi State for 92 km, to the south and southeast by Ondo State, and to the west by Osun State for 84 km. Ekiti is the 31st largest state in terms of area and the 30th most populous, with an estimated population of almost 3.3 million as of 2016 in Nigeria (14).

### Study design

The present study was a case-control study conducted on hypertensive patients attending the clinic at the Federal Teaching Hospital Ido (FETHI), Ido-Ekiti, Ekiti State, Nigeria from February, 2023 to August, 2023. Data collection was performed using a semi-structured questionnaire that included items on demographic characteristics such as age, sex, etc., and anthropometric parameters, such as weight and height. Sample size was calculated using the following formula: N=Z^2^pq/d^2^. The study population consisted of 155 subjects, comprising 83 hypertensive subjects and 72 sex- and age-matched healthy normotensive subjects as controls. Hypertensive subjects aged 18-60 years, with or without anti-hypertensive treatment, who provided consent were included in the study. Pregnant women, nursing mothers, individuals with other health conditions, those taking vitamins or mineral supplements, and those who did not provide consent were excluded from the study. The control subjects (normotensive) were apparently healthy individuals who had no history of high blood pressure and had no underlying health condition.

### Ethical approval

Ethical approval for sample collection was obtained from the Health Research Ethics Committee at the Federal Teaching Hospital Ido (FETHI), Ido-Ekiti, Ekiti State. Informed consent was obtained from each subject who participated in the study prior to sample collection. Additionally, the study was conducted in accordance with the Declaration of Helsinki.

### Sample collection and analysis

A total of 5 ml of blood samples were collected from each subject via venipuncture and dispensed into a plain bottle. The sample was allowed to clot for 1 h, after which the serum was separated by centrifugation at 16,087 x g for 5 min at room temperature. The serum was then carefully withdrawn into a pre-labeled tube and stored at -20˚C until analysis.

### Blood pressure measurements

Blood pressure was measured using a mercury sphygmomanometer (KENZ Model 605P, Suzuken Co., Ltd.). Participants were allowed to sit and rest in a quiet area for at least 15 min before the measurements were taken. Blood pressure was measured on the right arm with the arm relaxed and supported on a table at a 45˚ angle to the trunk. A total of three measurements taken at 5-min intervals were averaged for diastolic blood pressure (DBP) and systolic blood pressure (SBP). Variations of <5 mmHg were permitted. Based on their average SBP and DBP values, the study participants were divided into two groups (the hypertensive and non-hypertensive groups).

### Body mass index (BMI)

Weight measurements were performed using a weighing scale, height was measured using a meter rule, and BMI was calculated using the formula: Weight (kg)/height (m^2^).

### Biochemical parameters

Sodium and potassium levels were estimated using an ion selective electrolyte (ISE) analyzer branded Biolyte 2000 (BioCare Corporation) (15). Calcium, magnesium and phosphorus levels were estimated using spectrophotometry (722G VIS Spectrophotometer, Shanghai Drawell Scientific Instrument Co., Ltd.) (16). Uric acid, creatinine and urea levels were measured using a semi-auto chemistry analyzer (Mindray BA-88; serial no. BH7AB2710, Shenzhen Mindray Bio-Medical Electronics Co., Ltd.) based on the enzyme colorimetry (Uricase TBHBA), Jaffe and Berthelot methods, respectively (9). Vitamin D was estimated using enzyme-linked immunosorbent assay (ELISA) techniques (Elabscience Biotechnology Inc.) according to the manufacturer's instructions and as previously described (17).

### Statistical analysis

The results are presented using tables and charts as the mean ± standard deviation. Data analysis was conducted using SPSS software (version 23.0; IBM Corp.). Statistical significance was determined between groups using the Student's t-test, while Pearson's correlation analysis was used to determine the correlation between variables. A P-value <0.05 considered to indicate a statistically significant difference.

## Results

**Figure 1:**
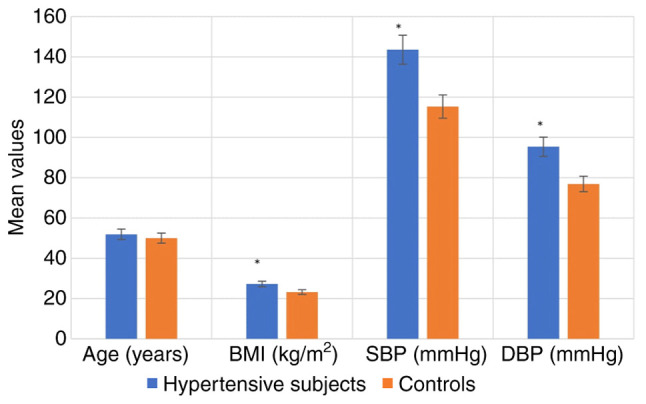
Anthropometric characteristics of the hypertensive subjects and controls. BMI, body mass index; DBP, diastolic blood pressure; SBP, systolic blood pressure.

**Figure 2:**
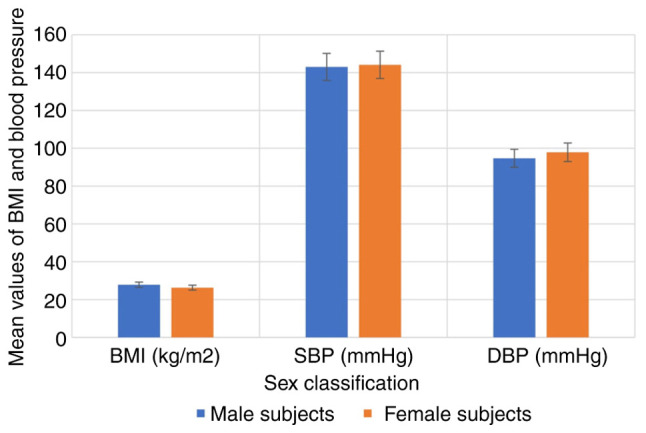
Anthropometric parameters of the hypertensive subjects as regards sex. BMI, body mass index; DBP, diastolic blood pressure; SBP, systolic blood pressure.

**Figure 3:**
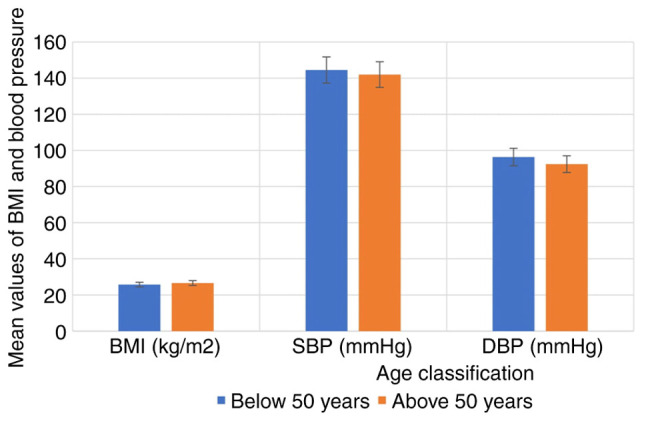
Anthropometric parameters of the hypertensive subjects according to age. BMI, body mass index; DBP, diastolic blood pressure; SBP, systolic blood pressure.

The anthropometric characteristics of the hypertensive subjects and controls are illustrated in Fig. 1. The results indicated that BMI, SBP and DBP were significantly higher in the hypertensive subjects compared with the controls (P<0.05). The anthropometric characteristics of the hypertensive subjects by sex are depicted in Fig. 2, while the anthropometric characteristics of the hypertensive subjects by age are depicted in Fig. 3. The results revealed that there were no significant differences in the BMI, SBP and DBP of the hypertensive subjects as regards age and sex (P>0.05).

The renal function parameters, electrolyte and vitamin D levels of the hypertensive subjects and the controls are presented in Table I. The findings revealed that the creatinine, urea, uric acid, magnesium, sodium, sodium/potassium ratio and sodium/calcium levels were significantly higher in the hypertensive subjects compared with the healthy controls (P<0.05). The vitamin D, calcium, potassium and phosphate levels were significantly lower in the hypertensive subjects compared with the healthy controls (P<0.05). There was no significant difference in the potassium/calcium ratio between the two groups (P>0.05). The renal function parameters, electrolyte and vitamin D levels of the hypertensive subjects based on sex are presented in Table II. The results revealed that there were no significant differences in all parameters studied based on sex (P>0.05). The renal function parameters, electrolyte and vitamin D levels of the hypertensive subjects according to age are demonstrated in Table III. The results indicated that there were no significant differences in all parameters studied based on age (P>0.05), apart from the phosphate levels, which were significantly higher in the subjects <50 years of age compared to those >50 years of age. The results of the correlation analysis between renal function parameters, electrolyte and vitamin D levels in the hypertensive subjects are presented in Table IV and Fig. S1, Fig. S2, Fig. S3, Fig. S4, Fig. S5 and Fig. S6. The results obtained revealed a significant correlation between the parameters examined and blood pressure. Creatinine (r=0.211, P=0.142), urea (r=0.088, P=0.543), uric acid (r=0.442, P=0.033), vitamin D (r=0.281, P=0.048), sodium (r=0.176, P=0.221) and potassium (r=0.342, P=0.020) exhibited positive correlations with SBP.

## Discussion

Obesity is a well-known significant risk factor for non-communicable diseases, such as hypertension. In the present study, BMI was found to be slightly higher in the hypertensive subjects compared to the control group. Previous systematic reviews and retrospective studies have consistently indicated that higher BMI values are associated with elevated blood pressure in adults (18-19). The underlying mechanisms are intricate and often interconnected. It has been reported that a BMI ≥23 serves as a risk factor for insulin resistance (20). Insulin resistance caused by the accumulation of excessive lipids, leads to lipid deposition in various tissues, such as blood vessels, triggering inflammation in the area. At the same time, excess fat triggers the secretion of pro-inflammatory cytokines, contributing to atherogenesis. Ultimately, these factors disrupt blood pressure regulation, potentially leading to elevated blood pressure or hypertension (21). In the present study, SBP and DBP were significantly higher in the hypertensive subjects compared with the control group. These findings are consistent with the findings of previous studies (22,23) that also found elevated SBP and DBP in hypertensive individuals. The increased pressure from hypertension can cause damage to blood vessels and impair organ function, increasing the risk of heart disease, stroke, chronic heart failure, kidney disease and other related conditions (24).

Clinical investigations of renal function using parameters such as uric acid, urea and creatinine are crucial for identifying renal dysfunction, diagnosing renal disease, monitoring disease progression and evaluating response to treatment. In the present study, urea, creatinine and uric acid levels were significantly higher in the hypertensive subjects compared with the control group. This finding aligns with the findings of previous studies (22,25-28). The increase in the serum creatinine concentration may be attributed to a decrease in creatinine clearance resulting from a decline in the glomerular filtration rate (GFR). This impairment may be a consequence of prolonged hypertension or a contributing factor to its development and progression (25). Furthermore, a reduction in renal blood flow leads to a decrease in GFR, subsequently reducing the distal tubular flow rate. This leads to increased urea reabsorption and decreased secretion, potentially explaining the elevated serum urea concentration (26). The elevated serum uric acid levels observed in the present study highlight its potential role in the pathophysiology of hypertension. This aligns with recent literature suggesting that hyperuricemia may contribute to hypertension through mechanisms such as renal vasoconstriction and endothelial dysfunction (27). Hyperuricemia can negatively affect cardiovascular and renal function by reducing endothelial nitric oxide levels and neuronal nitric oxide synthase in the macula densa of the kidney, thereby stimulating the RAAS (28). It may also trigger an inflammatory response, potentially causing glomerular damage, tubular ischemia, endothelial dysfunction, and the development of renal microvascular disease by stimulating vascular smooth muscle cell proliferation, ultimately leading to renal and systemic hypertension. Hyperuricemia is also linked to the presence of target organ damage in hypertensive patients (29).

In the present study, a significantly higher plasma sodium level was found in the hypertensive subjects compared to the controls. The reason for this difference may be attributed to variances in the cell membrane, an increase in a sodium transport inhibitor, a decreased sodium efflux rate constant and a reduction in the activity of the ouabain-sensitive component of the Na^+^-K^+^-ATPase pump (30). This finding supports the findings of previous studies that have reported an association between hypertension and elevated plasma sodium levels (30-32). There are several mechanistic explanations for the link between high sodium intake and blood pressure. These include enhanced reabsorption and retention of filtered sodium through the renal tubules, as well as activation of the brain RAAS. This system is suggested to increase blood pressure through angiotensin II and aldosterone, which promote local oxidative stress and activate the sympathetic nervous system (31). Furthermore, according to the ‘vasodysfunction theory’ of salt-induced hypertension, salt loading leads to inadequate decreases in systemic vascular resistance, resulting in increased blood pressure (33). Salt sensitivity, which varies among individuals, is considered to be a key factor in this process. Therefore, treatments for essential hypertension should focus on reducing plasma sodium levels by decreasing dietary salt intake, increasing sodium excretion, or inhibiting the RAAS (34).

The results of the present study revealed a significant decrease in plasma potassium levels among hypertensive subjects compared with the control group. This finding aligns with the findings of previous studies that have reported lower plasma potassium levels in both treated and untreated hypertensive individuals compared to controls (5,35,36). The most common cause of hypokalemia in hypertensive patients is the use of diuretic drugs. Both thiazide and loop diuretics increase urinary flow and sodium delivery through the collecting tubule, leading to renal potassium secretion (36). This secretion is further increased in the presence of diuretic-induced intravascular volume depletion and secondary aldosterone stimulation (37). Since potassium is essential for maintaining cardiac and vascular health, it is recommended to provide hypertensive patients on diuretic medications with an appropriate potassium supplement. This approach can improve outcomes and help prevent the development of vascular complications.

Magnesium plays a crucial role in regulating blood pressure by controlling vascular tone and reactivity. It acts as a calcium channel antagonist, which helps in the production of vasodilator prostacyclins and nitric oxide. Furthermore, magnesium helps maintain healthy endothelial function, regulates RAAS, relaxes blood vessels and influences how blood vessels respond to vasoactive agonists (38). In the present study, magnesium levels were significantly higher in hypertensive individuals compared to the controls. In vascular smooth muscle cells, magnesium functions by inhibiting transmembrane calcium transport and entry, thereby counteracting the effects of calcium. When magnesium levels are low, there is an increase in the intracellular free calcium concentration, leading to vascular contraction. Magnesium deficiency can also lead to endothelial dysfunction and increase in blood pressure, as the ability of the body to relax blood vessels and regulates the RAAS is impaired (39). On the other hand, elevated plasma magnesium levels have been observed in cases of both chronic and acute renal failure (40,41). The slightly higher mean serum magnesium level in hypertensive patients suggests a possible subclinical renal issue related to magnesium excretion (42). It is important to note that even though the mean magnesium level in these patients was elevated, it still fell within the normal reference range. This could explain why there were no obvious signs of renal dysfunction in these patients.

In addition to the interdependent effect of sodium and potassium, calcium and magnesium have also been implicated in the regulation of blood pressure. In the present study, calcium levels were found to be lower in the hypertensive subjects compared with the controls; however, the difference was not statistically significant. This finding is in agreement with the findings of previous studies (43,44). Low calcium levels lead to an increased activity of the parathyroid gland, which results in the release of parathyroid hormone (PTH). This hormone can then lead to the synthesis of calcitriol, either directly or mediated by PTH. Calcitriol, in turn, increases intracellular calcium in vascular smooth muscle cells causing vasoconstriction (43). The release of renin is stimulated by low extracellular calcium and PTH, activating the RAAS. PTH also enhances the synthesis of angiotensin II and aldosterone, further promoting vasoconstriction, increasing renal water reabsorption, and ultimately raising blood pressure (45). Therefore, increasing plasma calcium levels through vitamin D supplements can reduce blood pressure in hypertensive patients.

Vitamin D deficiency is an independent risk factor for high blood pressure and is also associated with an increased risk of cardiovascular mortality. The present study found that vitamin D levels were significantly lower in hypertensive subjects compared with the control group. Studies have suggested that individuals with lower levels of 1,25(OH)2D3 may be more likely to develop hypertension. This is due to the fact that a deficiency in 1,25(OH)2D3 can lead to the increased activity of the RAAS, both in the body as a whole and specifically in the kidneys (46). In a low 1,25(OH)2D3 setting, there is often an increase in the plasma renin concentration, which can raise sympathetic activity and increase intra-glomerular pressure. This can predispose individuals to hypertension, a decrease in the glomerular filtration rate, and eventual cardiovascular damage (47). Aside from the RAAS, vitamin D can also influence hypertension through several other mechanisms. Vitamin D plays a role in calcium homeostasis by promoting the production of calcium transporters, increasing calcium reabsorption in the kidneys, and stimulating the release of calcium from bones by osteoclasts (48). Consequently, a deficiency in vitamin D can lead to a decrease in plasma Ca^2+^ levels, triggering the secretion of PTH from the chief cells in the parathyroid gland to counteract this imbalance. Numerous epidemiological studies have demonstrated that elevated PTH levels are linked to a higher SBP and DBP, as well as an increased prevalence of hypertension in general (47). Several studies have reported a direct association between low plasma 25(OH)D concentrations and the risk of developing hypertension and related complications (47,49,50). Therefore, supplementation with vitamin D appears to be a promising therapeutic option for these patients.

The results of the present study revealed a significant correlation between renal function parameters, electrolyte and vitamin D levels in hypertensive subjects. Creatinine, urea, uric acid, vitamin D, sodium and potassium were all found to be positively correlated with SBP. Previous research has also shown a positive correlation between electrolytes, particularly sodium and potassium, and SBP in hypertensive subjects (18). Vitamin D deficiency has been shown to be associated with an increased risk of developing hypertension and cardiovascular diseases. Low serum vitamin D levels have been identified as a risk factor for cardiovascular disease and are linked to a higher incidence of hypertension, insulin resistance, obesity, metabolic syndrome, impaired fasting blood glucose levels, and dyslipidemia (51,52). Renal blood flow autoregulation may not completely protect the kidneys from the effects of moderately elevated systolic blood pressure. Early active management of moderate systolic hypertension in all adults could potentially reduce the risk of developing chronic kidney disease later in life. Maintaining healthy blood pressure levels is essential for preserving kidney function.

One limitation of the present study was the selection of participants from a specific region or hospital, which may not be representative of the entire hypertensive Nigerian population. Furthermore, the present study was a ‘snapshot’ of a single point in time, and may not capture changes in electrolytes, renal biomarkers and vitamin D levels over time during the course of treatment. Despite these limitations, the present study has key implications for clinical practice, such as identifying new targets for treatment or improving existing treatments, as well as setting a template for future research.

In conclusion, in the present study, electrolyte imbalance, renal dysfunction and vitamin D deficiency were observed in hypertensive subjects. The present study also revealed significant correlations between parameters that may affect renal function or be influenced by renal impairment. These findings emphasize the importance of assessing and monitoring these biochemical markers as they could improve prognosis, aid in early diagnosis of kidney damage, and assist in determining the optimal level of therapeutic interventions. Furthermore, the findings of the present study support the use of vitamin D supplementation as adjunctive therapy to manage hypertension, particularly in patients with vitamin D deficiency. Understanding the association between renal biomarkers, electrolytes imbalance and vitamin D levels may contribute to reducing the risk of developing cardiovascular disease and kidney damage, and may aid in the development of more effective treatment strategies to improve blood pressure control. However, further studies are required to focus on the analysis of additional biomarkers and to investigate hypertensive groups with different demographic characteristics. Furthermore, interventional design studies are warranted to evaluate the effects of vitamin D supplementation, electrolyte imbalance correction, and renal biomarkers-guided therapy on blood pressure control and cardiovascular health.

## Supplementary Material

Scatter plot illustrating a significant positive correlation between vitamin D and systolic blood pressure.

Scatter plot illustrating a significant positive correlation between uric acid concentration and diastolic blood pressure.

Scatter plot illustrating a significant positive correlation between the urea and creatinine concentration.

Scatter plot illustrating a significant positive correlation between the calcium and potassium concentration.

Scatter plot illustrating a significant positive correlation between the magnesium and calcium concentration.

Scatter plot illustrating a significant positive correlation between the potassium and uric acid concentration.

## Figures and Tables

**Table I:** Renal function parameters, electrolytes and vitamin D levels of the hypertensive subjects and controls.

Parameter	Hypertensive subjects (n=83)	Controls (n=72)	P-value
Creatinine (µmol/l)	129.91±8.74	96.04±8.44	0.001^a^
Urea (mmol/l)	6.87±1.06	4.81±0.89	0.006^a^
Uric acid (µmol/l)	329.84±64.42	294.30±55.74	0.039^a^
Magnesium (mg/dl)	1.19±0.29	0.97±0.28	0.044^a^
Phosphate (mmol/l)	1.12±0.51	1.66±0.62	0.001^a^
Sodium (mmol/l)	141.36±3.79	135.74±3.55	0.001^a^
Potassium (mmoll)	4.01±0.67	4.26±0.51	0.017^a^
Calcium (mmol/l)	2.12±0.31	2.47±0.33	0.012^a^
Vitamin D (ng/ml)	29.54±2.59	42.35±6.09	0.001^a^
Sodium/potassium ratio	35.25±2.26	31.86±2.35	0.001^a^
Sodium/calcium ratio	66.68±2.89	54.96±2.67	0.001^a^
Potassium/calcium ratio	1.89±0.16	1.72±0.34	0.179

^a^Indicates a statistically significant difference (P<0.05).

**Table II:** Renal function parameters, electrolytes and vitamin D levels of the hypertensive subjects as regards sex.

Parameter	Male subjects (n=39)	Female subjects (n=44)	P-value
Creatinine (µmol/l)	134.34±11.20	126.10±7.01	0.252
Urea (mmol/l)	5.25±1.52	5.83±1.67	0.213
Uric acid (µmol/l)	301.25±70.66	284.50±72.50	0.514
Magnesium (mg/dl)	1.02±0.24	1.15±0.31	0.215
Phosphate (mmol/l)	0.98±0.49	1.26±0.49	0.069
Sodium (mmol/l)	136.75±3.86	135.92±3.70	0.468
Potassium (mmol/l)	4.13±0.79	4.07±0.53	0.802
Calcium (mmol/l)	2.54±0.31	2.24±0.30	0.177
Vitamin D (ng/ml)	30.04±15.26	27.70±15.05	0.111
Sodium/potassium ratio	33.11±2.16	33.39±2.41	0.672
Sodium/calcium ratio	53.84±3.91	60.68±3.84	0.098
Potassium/calcium ratio	1.63±0.12	1.82±0.21	0.321

**Table III:** Renal function parameters, electrolytes and vitamin D levels of the hypertensive subjects as regards age.

Parameter	<50 years of age (n=40)	>50 years of age (n=43)	P-value
Creatinine (µmol/l)	134.49±15.03	124.02±11.24	0.053
Urea (mmol/l)	5.73±1.59	5.07±1.53	0.104
Uric acid (µmol/l)	313.04±54.57	289.37±53.30	0.345
Magnesium (mg/dl)	1.09±0.23	1.08±0.31	0.884
Phosphate (mmol/l)	1.33±0.50	0.92±0.39	0.007^a^
Sodium (mmol/l)	136.52±3.58	136.35±3.85	0.886
Potassium (mmol/l)	4.10±0.45	4.17±0.82	0.709
Calcium (mmol/l)	2.39±0.33	2.53±0.24	0.167
Vitamin D (ng/ml)	41.05±5.16	44.97±7.06	0.396
Sodium/potassium ratio	33.29±3.01	32.69±2.97	0.459
Sodium/calcium ratio	57.12±3.78	53.89±4.02	0.212
Potassium/calcium ratio	1.72±0.15	1.65±0.16	0.623

^a^Indicates a statistically significant difference (P<0.05).

**Table IV:** Correlation between renal function parameters, electrolytes and vitamin D levels in hypertensive subjects.

		SBP	DBP	CRT	Urea	UA	Mg	Ca	Na	K	VitD
SBP	Pearson's correlation		0.608^b^	0.211	0.088	0.442	-0.001	-0.065	0.176	0.342	0.281^a^
	Sig. (2-tailed)		0.000	0.142	0.543	0.033^a^	0.996	0.655	0.221	0.020	0.048
DBP	Pearson's correlation	0.608^b^		0.085	0.134	0.442	0.004	-0.083	-0.083	0.248	-0.115
	Sig. (2-tailed)	0.001		0.558	0.353	0.033^a^	0.981	0.568	0.566	0.082	0.425
CRT	Pearson's correlation	0.211	0.085		0.281^a^	0.248	-0.039	-0.268	-0.024	0.038	-0.116
	Sig. (2-tailed)	0.142	0.558		0.048	0.082	0.787	0.060	0.870	0.791	0.421
Urea	Pearson's correlation	0.088	0.134	0.281^a^		0.064	-0.049	-0.004	-0.023	0.193	-0.012
	Sig. (2-tailed)	0.543	0.353	0.048		0.661	0.734	0.980	0.873	0.179	0.936
UA	Pearson's correlation	0.442	0.129	0.248	0.064		0.038	0.108	-0.023	0.306^a^	0.122
	Sig. (2-tailed)	0.033^a^	0.371	0.082	0.661		0.793	0.457	0.875	0.031	0.398
Mg	Pearson's correlation	-0.001	0.004	-0.039	-0.049	0.038		0.292^a^	0.269	0.095	-0.108
	Sig. (2-tailed)	0.996	0.981	0.787	0.734	0.793		0.040	0.059	0.510	0.455
Ca	Pearson's correlation	-0.065	-0.083	-0.268	-0.004	0.108	0.292^a^		0.116	0.330^a^	0.124
	Sig. (2-tailed)	0.655	0.568	0.060	0.980	0.457	0.040		0.421	0.019	0.392
Na	Pearson's correlation	0.176	-0.083	-0.024	-0.023	-0.023	0.269	0.116		0.192	-0.156
	Sig. (2-tailed)	0.221	0.566	0.870	0.873	0.875	0.059	0.421		0.182	0.279
K	Pearson's correlation	0.342	0.248	0.038	0.193	0.306^a^	0.095	0.330^a^	0.192		0.001
	Sig. (2-tailed)	0.020	0.082	0.791	0.179	0.031	0.510	0.019	0.182		0.995
Vit.D	Pearson's correlation	0.281^a^	0.115	-0.116	-0.012	0.122	-0.108	0.124	-0.156	0.001	
	Sig. (2-tailed)	0.048	0.425	0.421	0.936	0.398	0.455	0.392	0.279	0.995	

^a^Correlation is statistically significant (P<0.05; 2-tailed;

^b^correlation is statistically significant (P<0.01; 2-tailed). UA, uric acid; CRT, creatinine; Mg, magnesium; Ca, calcium; Na, sodium; K, potassium; VitD, vitamin D; SBP, systolic blood pressure; DBP, diastolic blood pressure.

## Data Availability

The data generated in the present study may be requested from the corresponding author.
