# Supplementary material for: Correlation of renal biomarkers, electrolyte imbalances and vitamin D levels in hypertensive subjects
Source: Med Int (Lond). 2025 Feb 5;5(2):20. doi: 10.3892/mi.2025.219 (PMC11843082; doi:10.3892/mi.2025.219)

Figure S1. Scatter plot illustrating a significant positive correlation between vitamin D and systolic blood pressure.

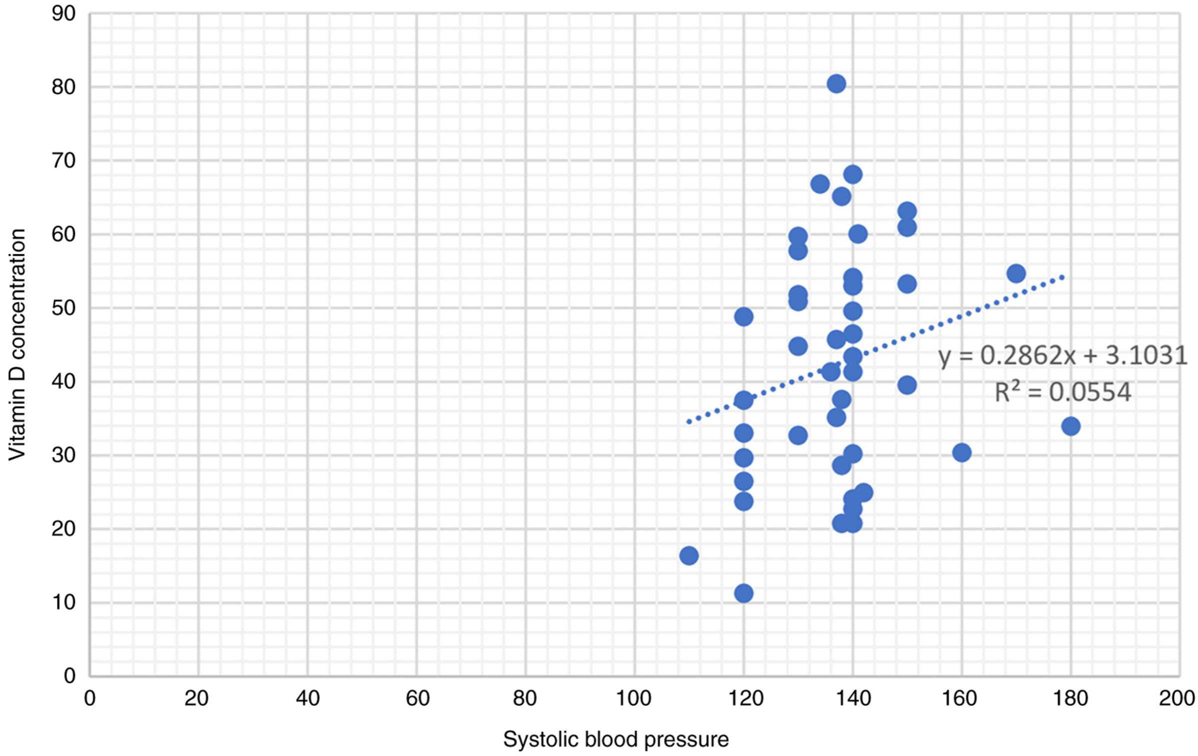

Figure S2. Scatter plot illustrating a significant positive correlation between uric acid concentration and diastolic blood pressure.

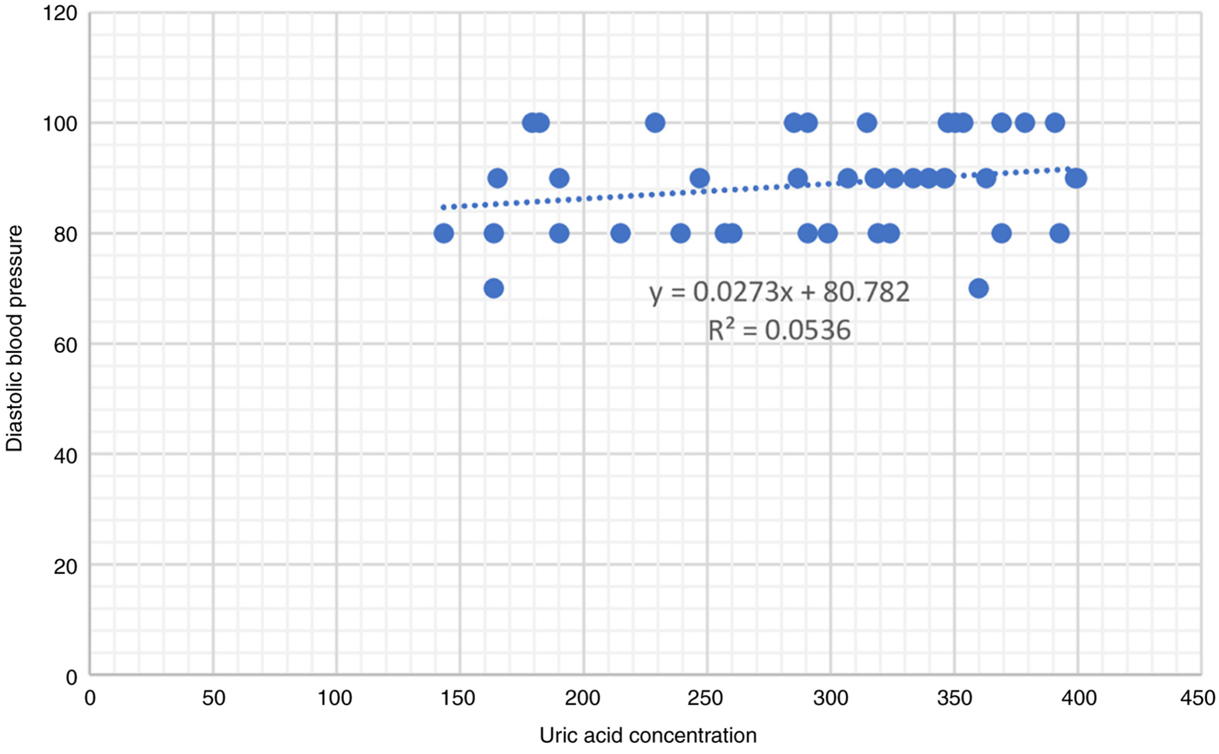

Figure S3. Scatter plot illustrating a significant positive correlation between the urea and creatinine concentration.

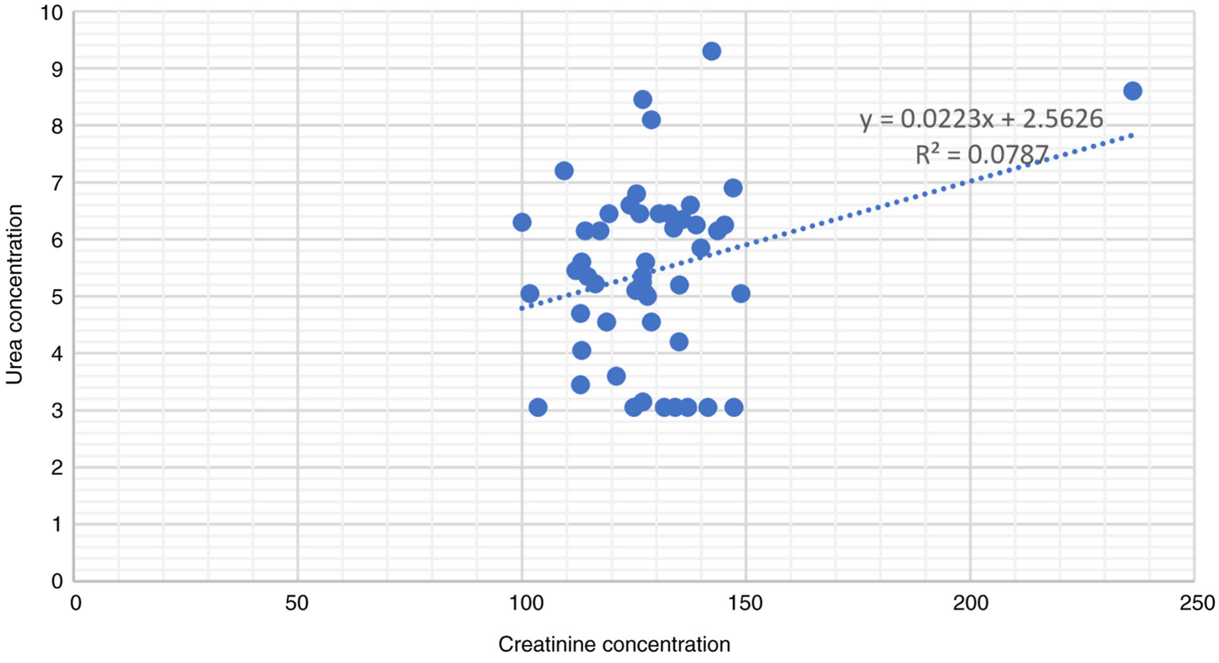

Figure S4. Scatter plot illustrating a significant positive correlation between the calcium and potassium concentration.

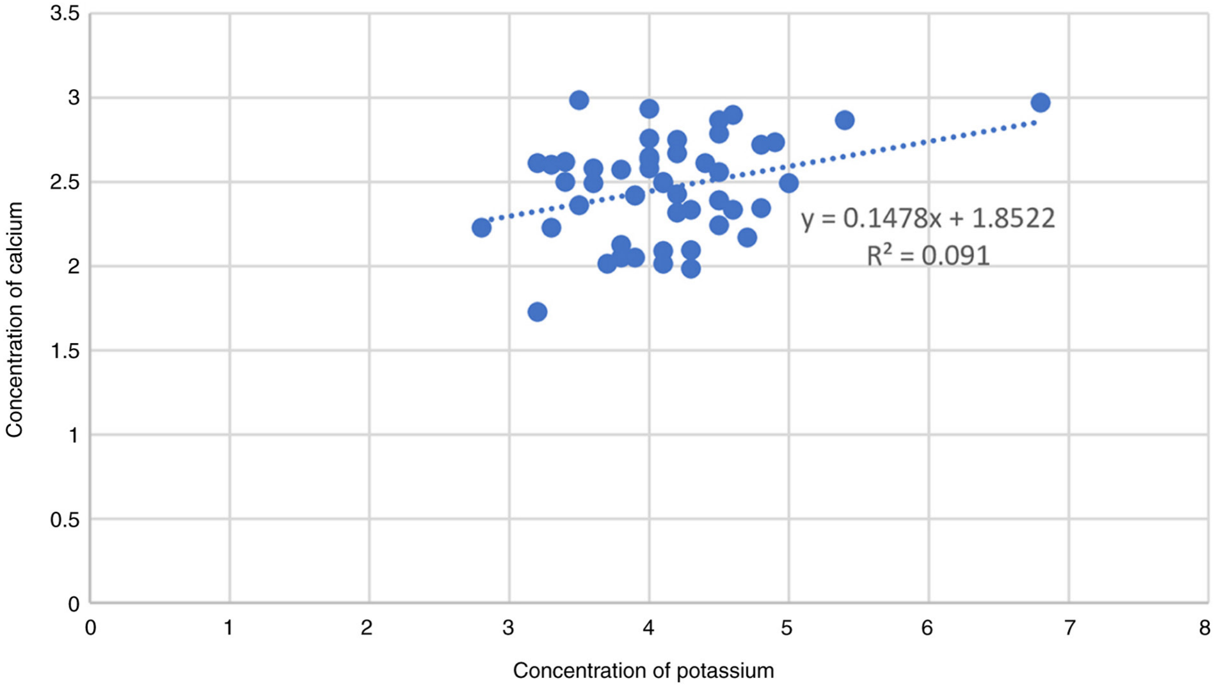

Figure S5. Scatter plot illustrating a significant positive correlation between the magnesium and calcium concentration.

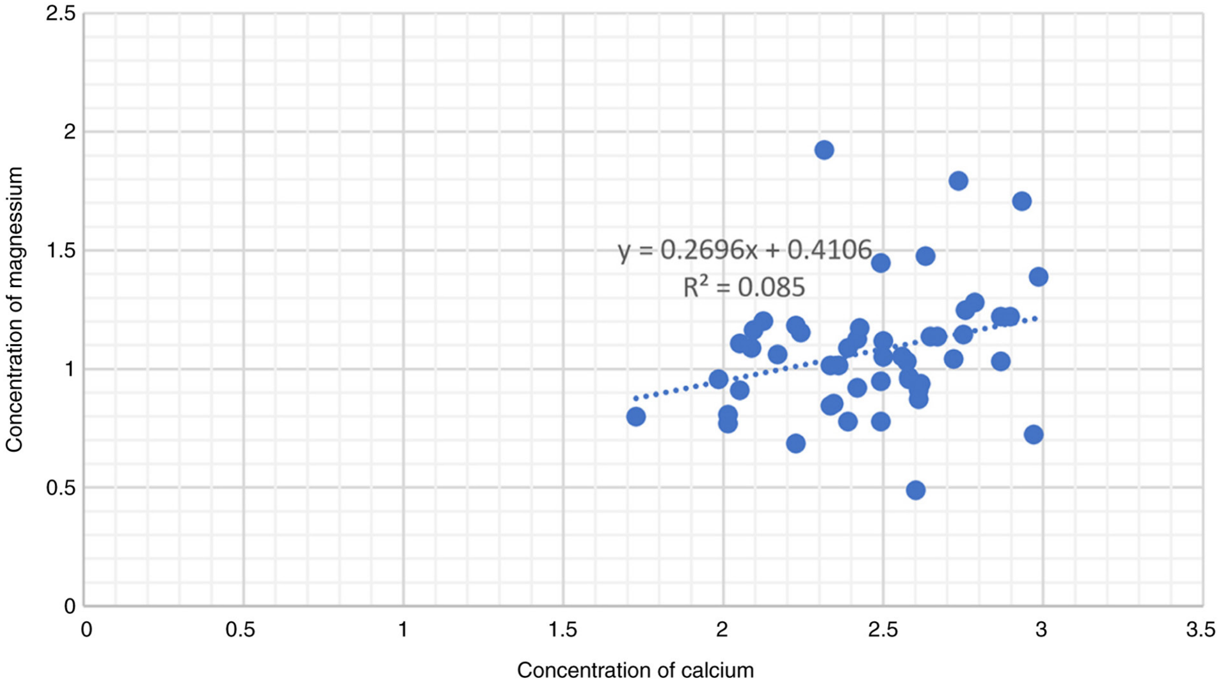

Figure S6. Scatter plot illustrating a significant positive correlation between the potassium and uric acid concentration.

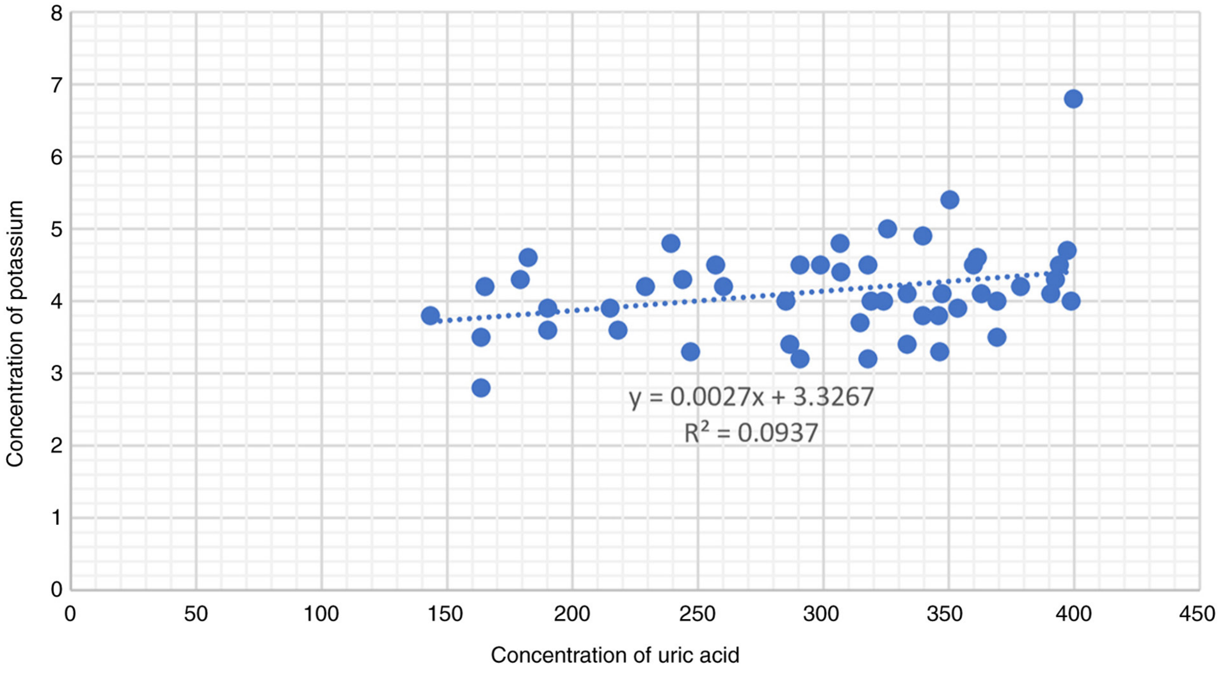

Supplement: Scatter plot illustrating a significant positive correlation between vitamin D and systolic blood pressure. [file Supplementary_Data.pdf]
